# Chloride at the spotlight—but not alone: towards a multi-ionic diuretic strategy

**DOI:** 10.1093/ckj/sfag119

**Published:** 2026-05-05

**Authors:** Marco Montomoli, Maria Ines Gil Martins Roxo, Miguel González-Rico, Julio Núñez, Gonzalo Núñez-Marín, Belén Aguilar Uriarte, Rafael De La Espriella, María Jesús Puchades, José L Górriz

**Affiliations:** Department of Nephrology, Clinical University Hospital of Valencia; INCLIVA Biomedical Research Institute, University of Valencia, Valencia, Spain; Department of Nephrology, West Lisbon Local Health Unit, Lisbon, Portugal; Department of Nephrology, Clinical University Hospital of Valencia; INCLIVA Biomedical Research Institute, University of Valencia, Valencia, Spain; Department of Cardiology, Clinical University Hospital of Valencia; INCLIVA Biomedical Research Institute; University of Valencia, Valencia, Spain; Department of Cardiology, Clinical University Hospital of Valencia, Valencia, Spain; Department of Nephrology, Clinical University Hospital of Valencia, University of Valencia, Valencia, Spain; Department of Cardiology, Clinical University Hospital of Valencia; INCLIVA Biomedical Research Institute, Valencia, Spain; Department of Nephrology, Clinical University Hospital of Valencia, University of Valencia, Valencia, Spain; Department of Nephrology, Clinical University Hospital of Valencia; INCLIVA Biomedical Research Institute; University of Valencia, Valencia, Spain

**Keywords:** chloride, chronic kidney disease, diuretic resistance, electrolyte disorders, heart failure, magnesium, metabolic alkalosis, potassium, volume assessment

## Abstract

Diuretic resistance in heart failure and the cardiorenal syndrome is traditionally managed by escalating diuretic doses, yet this approach often fails to address the underlying pathophysiology. Emerging evidence challenges the sodium-centred paradigm and identifies chloride as a key determinant of diuretic responsiveness, tightly interconnected with potassium, magnesium, and acid–base balance. Disturbances in this multi-ionic network promote maladaptive tubular responses, neurohormonal activation, and persistent sodium retention, ultimately leading to diuretic resistance. Clinical and mechanistic data consistently show that hypochloraemia, often accompanied by hypokalaemia and metabolic alkalosis, is associated with impaired natriuretic efficiency and worse outcomes. Through a representative clinical case, we illustrate how a physiology-guided, multi-ionic strategy—focused on identifying and correcting the specific biochemical drivers of resistance rather than intensifying diuretics—can restore diuretic response and achieve effective decongestion. Diuretic resistance should not be viewed as a failure of dose, but as a failure of understanding. A personalized, multi-ionic approach that integrates chloride, potassium, magnesium, and acid–base status offers a more coherent and effective framework for managing congestion in heart failure.

## Graphical Abstract

**Graphical Abstract:**
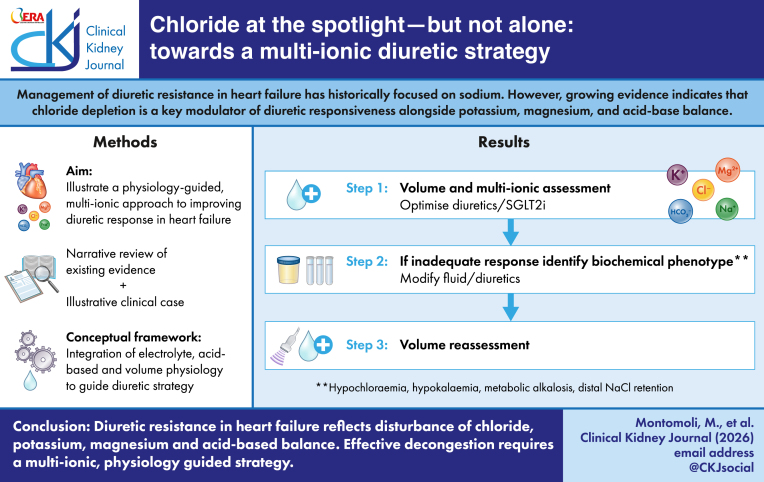

## CLINICAL CASE: MULTI-IONIC CLUES IN REFRACTORY CONGESTION

A 74–year–old man with heart failure (HF) with reduced ejection fraction (left ventricular ejection fraction 32% of ischaemic origin), arterial hypertension, permanent atrial fibrillation, and chronic kidney disease (CKD) G3bA3 was admitted with progressive dyspnoea (New York Heart Association class III), jugular venous distension, massive peripheral oedema, and a 3–kg weight gain despite guideline–directed medical therapy and oral loop diuretics.

A structured multimodal congestion assessment confirmed advanced systemic and pulmonary congestion: echocardiography confirmed heart failure with reduced ejection fraction (HFrEF) (left ventricular ejection fraction 32%) with right ventricular dilatation, tricuspid annular plane systolic excursion 14 mm, moderate tricuspid regurgitation, and elevated systolic pulmonary artery pressure. Venous Excess Ultrasound Score (VExUS) grade 3 comprised inferior vena cava (IVC) 24 mm with <10% inspiratory collapse, portal vein pulsatility fraction >50%, and severe intrarenal venous flow (IRVF) reversal (monophasic diastolic pattern), and whole–body bioimpedance revealed increased extracellular water with an elevated extracellular/intracellular water ratio, indicating marked extravascular fluid overload with relatively preserved intravascular volume.

Baseline laboratory tests (Table 1) showed markedly elevated N-terminal pro-B-type natriuretic peptide (NT–proBNP) and CA–125, serum sodium 139 mmol/l, mild hypochloraemia (chloride 94 mmol/l), metabolic alkalosis (bicarbonate 31 mmol/l, arterial pH 7.48), borderline hypokalaemia (3.3 mmol/l), normal magnesium, and serum creatinine 1.8 mg/dl with severe albuminuria (albumin-to-creatinine ratio (ACR) greater than 350 mg/g), consistent with HF–related congestion on a background of CKD. From admission to 48 h, the patient received intravenous furosemide 250 mg/day with poor response (urine output 900 ml/day and no weight loss), and hydrochlorothiazide 50 mg/day was added at 24 h to intensify sequential nephron blockade, reflecting local availability and previous outpatient use, although thiazide–like agents would generally provide more potent distal blockade at this level of kidney function.

**Table 1:** On admission laboratory values.

Parameter	Result	Normal range
NT-proBNP	12 500 pg/ml ↑	<300 pg/ml
CA-125	162 U/ml ↑	<35 U/ml
Na⁺	139 mmol/l	135–145 mmol/l
Cl⁻	94 mmol/l ↓	98–107 mmol/l
HCO₃⁻	31 mmol/l ↑	22–28 mmol/l
K⁺	3.3 mmol/l ↓	3.5–5.1 mmol/l
Mg⁺	1.03 mmol/l	0.85–1 .10 mmol/l
pH	7.48 ↑	7.35–7.45
Creatinine	1.8 mg/dl ↑	0.7–1.3 mg/dl
ACR	>350 mg/g (A3)	<30 mg/g

At 48 h (end of the initial phase), persistent VExUS grade 3 with unchanged IVC/portal vein pulsatility/IRVF patterns, oliguria, and worsening electrolyte disturbances: serum chloride 89 mmol/l (from baseline 94 mmol/l), potassium 3.3 mmol/l, bicarbonate 34 mmol/l, arterial pH 7.50, and spot urinary chloride <20 mmol/l. This profile reflects combined mechanisms—early diuretic-mediated chloride wasting followed by dilutional hypochloraemia from persistent congestion with avid tubular reabsorption (low urinary Cl⁻)—compatible with chloride-depletion metabolic alkalosis and diuretic resistance (Table 2). Serum bicarbonate was measured immediately prior to initiating the multi-ionic strategy: hydrochlorothiazide was withdrawn and oral acetazolamide and potassium chloride were started, while intravenous furosemide 250 mg/day was continued; in addition, a low–volume infusion of hypertonic saline (3% NaCl, 15 ml/h for 24 h; total 360 ml) was administered under close monitoring in a monitored unit as a nonstandard but physiologically targeted adjunct to correct profound hypochloraemia and restore distal chloride delivery.

**Table 2:** Clinical and laboratory parameters during the follow-up.

Time point	Time from admission	Time from adjustment	Diuresis (ml/day)	Weight change (kg)	S. Cl⁻ (mmol/l)	K⁺ (mmol/l)	U. Cl⁻ (mmol/l)	pH	Creatinine (mg/dl)	NT-proBNP (pg/ml)	VExUS score
Pre-adjustment (end of initial phase)	48 h	–	900	0 (vs. admission)	89	3.3	<20	7.50	2.0	–	3
Early response	∼72 h	24 h (Day 1)	2600	−2.5	100	3.9	45	7.42	1.9	8300	1
Continued response	∼96 h	48 h (Day 2)	3000	−4.0	102	4.2	65	7.40	1.7	–	0
Hospital discharge	Day 5	Day 3	2400	−4.5	103	4.3	72	7.39	1.6	6900	0
Early postdischarge follow-up	Day 12 (7 days postdischarge)	–	2200	−5.0 (total)	104	4.4	75	7.40	1.6	5800	0

S. Cl⁻: serum chloride; U. Cl⁻ : urine chloride; K⁺: serum potassium.

Within 24–48 h, urine output rose to 2600–3000 ml/day, serum and urinary chloride normalized, metabolic alkalosis resolved, potassium levels returned to the normal range, and the VExUS score improved from 3 to 0: IVC 16 mm with >50% collapse, normal portal vein flow (<50%), and normal IRVF (continuous triphasic). By discharge and at early postdischarge follow–up, the patient remained clinically euvolaemic on a simplified regimen including loop diuretics, acetazolamide, and a mineralocorticoid receptor antagonist, with stable kidney function and sustained correction of hypochloraemia, hypokalaemia, and metabolic alkalosis (Tables 1 and 2).

## INTRODUCTION: WHY PAY ATTENTION TO CHLORIDE?

**Figure 1:**
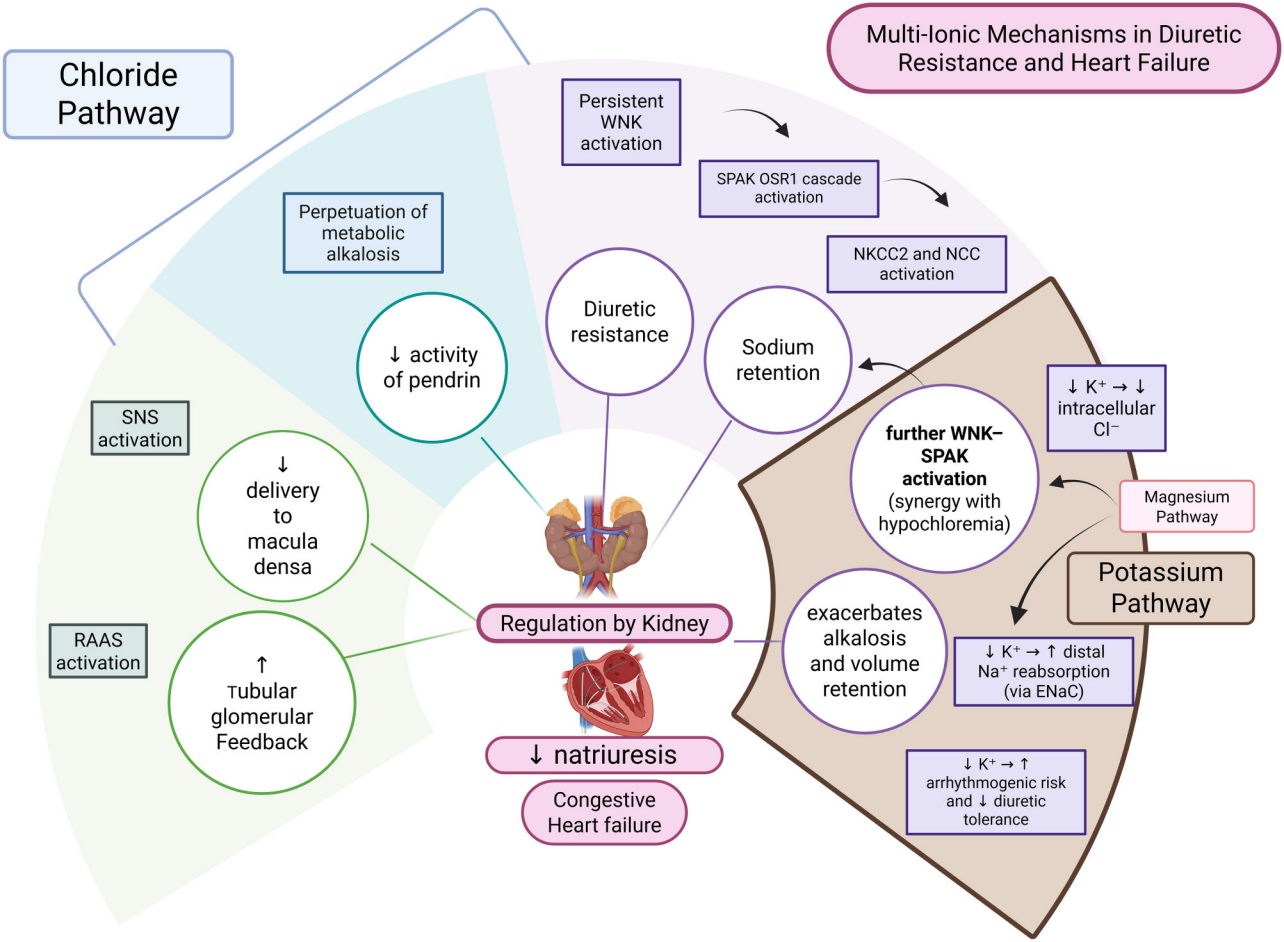
Multi-ionic mechanisms underlying diuretic resistance in heart failure. This schematic summarises the integrated role of chloride, potassium, and acid–base disturbances in the development of diuretic resistance in heart failure. Chloride depletion activates multiple maladaptive pathways, including reduced macula densa chloride delivery with consequent RAAS and sympathetic nervous system activation, impaired tubuloglomerular feedback, and persistent activation of the WNK–SPAK–OSR1 signalling cascade, leading to enhanced NKCC2 and NCC activity and sodium retention. Concurrently, chloride depletion limits pendrin-mediated bicarbonate secretion in the cortical collecting duct, perpetuating metabolic alkalosis and further reducing natriuretic efficiency.Potassium depletion amplifies these mechanisms by lowering intracellular chloride, thereby reinforcing WNK–SPAK activation, increasing distal sodium reabsorption (including ENaC activity), and exacerbating alkalosis and volume retention. Together, these interdependent chloride- and potassium-mediated pathways converge at the renal level to reduce effective natriuresis, sustain congestion, and promote diuretic resistance in heart failure.

HF and diuretic resistance are common and clinically challenging, demanding more effective and physiologically grounded decongestive strategies. Traditionally, renal sodium handling has been viewed as the primary determinant of diuretic response, but accumulating evidence indicates that chloride plays a pivotal and active role in the pathophysiology of HF and diuretic resistance and may be a relevant therapeutic target to overcome refractoriness (Fig. 1) [1].

Chloride homeostasis is tightly interconnected with potassium (K⁺) balance and acid–base status, forming an integrated system that modulates renal sodium transport, neurohormonal activation, and diuretic efficacy; disturbances in any component amplify maladaptive responses, favouring sodium retention, metabolic alkalosis, and progressive diuretic resistance. In this narrative review, the pathophysiological role of chloride in HF-related diuretic resistance and its therapeutic implications are examined, integrating emerging evidence on potassium depletion, metabolic alkalosis, and related electrolyte abnormalities within a multi-ionic framework.

## FUNDAMENTAL PHYSIOLOGY OF CHLORIDE AND RENAL HANDLING

Chloride (Cl⁻) is the main extracellular anion, essential for osmotic balance, vascular tone, membrane excitability, and acid–base homeostasis; plasma concentrations are tightly regulated, whereas much lower intracellular levels reflect active transport rather than passive equilibrium [2]. The kidney reclaims nearly all filtered chloride by coordinated passive and active mechanisms, thereby maintaining systemic homeostasis, and chloride also acts as a signalling ion that modulates sodium transport and neurohormonal tone [2, 3].

Basolateral chloride transport in the distal nephron is crucial for salt handling and diuretic sensitivity. ClC-K2 (the human ClC-Kb orthologue) is the predominant basolateral chloride channel in the thick ascending limb, distal convoluted tubule, and intercalated cells, and loss of function produces a Bartter-like phenotype with salt wasting, hypokalaemia, metabolic alkalosis, and reduced diuretic response; in humans, CLCNKB mutations reproduce this phenotype, underscoring the importance of basolateral chloride for Na⁺–K⁺–2Cl⁻ cotransporter (NKCC2)/Na⁺–Cl⁻ cotransporter (NCC) function and diuretic sensitivity and foreshadowing adaptive changes during chronic diuretic therapy [4, 5].

## CHLORIDE–POTASSIUM CROSSTALK AND THE WNK–SPAK–NCC PATHWAY

**Figure 2:**
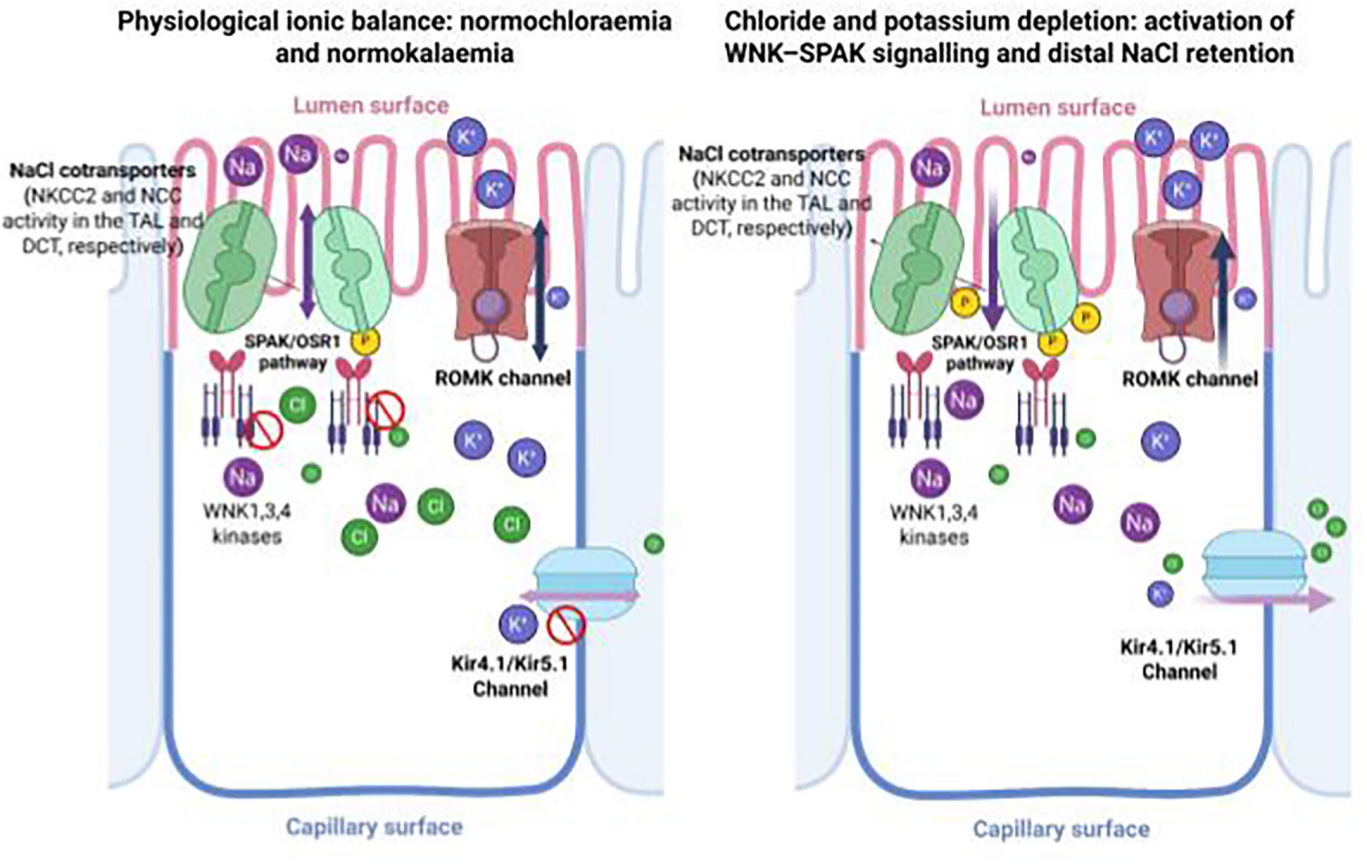
Chloride–potassium cross-talk and regulation of distal NaCl transport via the WNK–SPAK–NCC pathway. Under normochloremic and normokalemic conditions, intracellular chloride binds to WNK kinases (WNK1/WNK3), maintaining them in an inactive state and limiting SPAK/OSR1 phosphorylation and NCC activity in the distal convoluted tubule (DCT), thereby supporting effective natriuresis.In contrast, hypochloremia reduces intracellular Cl⁻ availability, relieving WNK inhibition and activating the WNK–SPAK–OSR1 cascade, resulting in increased NCC-mediated NaCl reabsorption and impaired natriuresis. Hypokalaemia further amplifies this process by hyperpolarising the basolateral membrane, promoting Cl⁻ efflux and further lowering intracellular chloride, which enhances WNK–SPAK signalling and NCC activation.Together, these mechanisms illustrate how combined chloride and potassium depletion promotes maladaptive distal NaCl reabsorption, contributing to diuretic resistance.

Intracellular chloride is a key regulator of distal sodium handling via the with-no-lysine (K) kinases (WNK)–STE20/SPS1-related proline/alanine-rich kinase (SPAK) signalling pathway (Fig. 2). Reductions in intracellular Cl⁻—as occur during loop or thiazide therapy—relieve WNK inhibition, leading to SPAK/oxidative stress-responsive kinase 1 (OSR1) activation and increased NKCC2 and NCC activity in distal nephron segments, an adaptive response that enhances distal NaCl reabsorption and progressively limits natriuretic efficacy [6–8].

Cl⁻ depletion seldom occurs in isolation. Diuretic-induced hypokalaemia further lowers intracellular chloride by promoting basolateral membrane hyperpolarization and chloride efflux, thereby amplifying WNK–SPAK–NCC signalling [9, 10]. Chronic K⁺ loss is associated with structural and functional remodelling of the distal nephron, including upregulation of sodium transporters and Na⁺/K⁺-ATPase, which increases the kidney’s capacity for compensatory sodium reabsorption and counteracts diuretic effects [11, 12].

At the basolateral membrane, reduced Kir4.1/Kir5.1 activity during hypokalaemia hyperpolarizes the cell, favours Cl⁻ efflux, further lowers intracellular chloride, and thereby relieves WNK inhibition, activates SPAK/OSR1, and stimulates NCC-mediated NaCl reabsorption—mechanisms mirrored in Gordon syndrome and hypokalaemic nephropathy [9, 10]. Chronic loop diuretic therapy with accompanying potassium loss additionally promotes hypertrophy and hyperplasia of distal nephron segments, reinforcing upregulation of NCC, NKCC2, and Na⁺/K⁺-ATPase and contributing to diuretic resistance [11, 12].

Metabolic alkalosis further increases distal H⁺ secretion and enhances renal outer medullary potassium channel (ROMK)-mediated K⁺ wasting, thereby reducing natriuretic efficiency [12]. A recent human study showed that an acute oral K⁺ load increases natriuresis in healthy individuals, underscoring that effective NaCl excretion depends on tightly coordinated K⁺–Cl⁻ regulation within the distal nephron and highlighting the inherently multi-ionic nature of diuretic responsiveness [13].

## MAGNESIUM AS A PERMISSIVE MODULATOR OF DISTAL TUBULAR HANDLING

In the setting of chronic diuretic exposure, magnesium (Mg²⁺) deficiency frequently accompanies Cl⁻ and K⁺ depletion and further impairs natriuretic efficiency by enhancing distal potassium secretion and sustaining WNK–SPAK activation [12, 14, 15]. Hypomagnesaemia lowers the threshold for K⁺ wasting by disinhibiting ROMK channels and facilitating epithelial sodium channel (ENaC)-dependent sodium reabsorption, thereby exacerbating hypokalaemia, metabolic alkalosis, and maladaptive distal Na⁺ retention [16, 17].

Beyond its effects on K⁺ handling, Mg²⁺ modulates distal Na⁺ transport by influencing NCC abundance and activity, partly through neural precursor cell expressed, developmentally down-regulated 4-2 (NEDD4-2)–dependent ubiquitination, and NEDD4-2 acts as a molecular tag targeting transport proteins such as NCC for degradation and thus influencing NaCl reabsorption and electrolyte balance [15, 18]. Within this integrated framework, hypomagnesaemia functions primarily as a permissive and amplifying factor rather than an independent driver of diuretic resistance, lowering the threshold at which Cl⁻ depletion and hypokalaemia translate into persistent sodium retention.

## CHLORIDE, RAAS ACTIVATION, AND TUBULOGLOMERULAR FEEDBACK

At the macula densa, distal luminal chloride concentration (rather than systemic hypochloraemia per se) is the principal signal driving tubuloglomerular feedback and renin release, with reduced NaCl delivery leading to enhanced prostaglandin E2 release, afferent arteriolar vasodilation, and increased renin secretion [19–21]. Experimental models with impaired distal chloride transport or pharmacologic inhibition of macula densa NaCl sensing demonstrate marked upregulation of renin and activation of the renin–angiotensin–aldosterone system (RAAS), underscoring that chloride, rather than sodium per se, is the key determinant of this pathway [19]. Chronic attenuation of macula densa chloride sensing, as may occur with sustained loop or thiazide therapy and chloride depletion, thus favours persistent RAAS activation, efferent arteriolar constriction, and enhanced proximal and distal sodium chloride reabsorption, which together reduce effective diuretic natriuresis [3].

Downstream, sustained aldosterone exposure in this setting increases ENaC expression and activity in the collecting duct, promoting electrogenic sodium reabsorption coupled to renal potassium and proton secretion and thereby contributing to hypokalaemia and metabolic alkalosis [14]. Aldosterone excess also modulates intercalated cell transport, with experimental data indicating that chronic hyperaldosteronism downregulates the apical Cl⁻/HCO₃⁻ exchanger pendrin and alters bicarbonate handling [22]. In hypochloraemic metabolic alkalosis with low urinary chloride (previously termed ‘chloride-sensitive’), reduced luminal chloride in the cortical collecting duct further limits pendrin-mediated bicarbonate secretion, thereby sustaining bicarbonate retention and alkalosis despite overall volume expansion [23].

Hypokalaemia, which frequently accompanies diuretic-induced chloride depletion, amplifies these processes by lowering luminal and intracellular potassium, favouring NH_4_^+^ transport via the thick ascending limb Na–K–2Cl cotransporter and stimulating renal ammoniagenesis [11, 12]. The resulting increase in NH_4_^+^ generation and recycling facilitates net bicarbonate production and reabsorption, reinforcing metabolic alkalosis and making its correction more difficult without simultaneous restoration of chloride and potassium [24]. Within this integrated framework, hypochloraemia, RAAS activation, ENaC upregulation, pendrin dysfunction, and enhanced ammoniagenesis constitute a self-reinforcing loop that promotes sodium retention, potassium wasting, and alkalosis, providing a strong mechanistic rationale for multi-ionic interventions targeting chloride and potassium repletion alongside RAAS and ENaC modulation [3].

Dietary and therapeutic factors can disrupt this chloride-sensitive feedback loop. Excessive sodium restriction or intensive diuretic therapy may disproportionately reduce chloride and potassium availability, amplifying RAAS activation, vasoconstriction, metabolic alkalosis, and potassium wasting [5, 25]. Modulation of tubular transporters and hormone receptor signalling also reshapes this integrated network: inhibition of proximal sodium–solute cotransport increases distal Na⁺ and Cl⁻ delivery, modifies macula densa signalling, and attenuates WNK–SPAK–NCC activation, while mineralocorticoid receptor antagonism reduces ENaC-mediated sodium reabsorption, limits K⁺ wasting, and may directly modulate WNK–SPAK signalling, thereby improving natriuretic efficiency and altering the Cl⁻–K⁺ balance [26–31].

## CHLORIDE DEPLETION, PENDRIN DYSFUNCTION, AND METABOLIC ALKALOSIS

**Figure 3:**
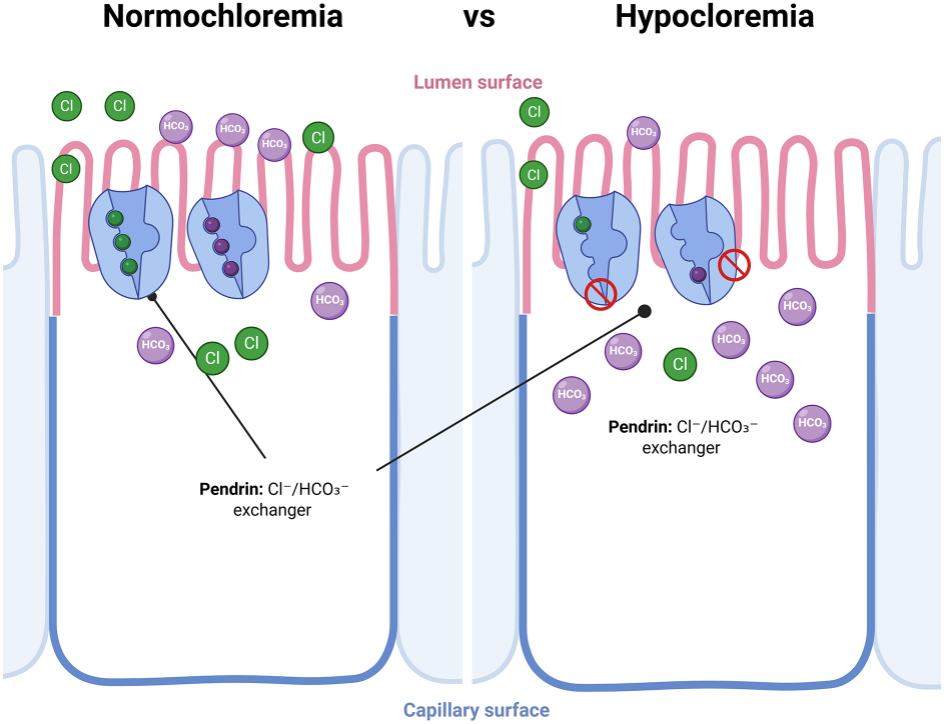
Chloride depletion, pendrin dysfunction, and metabolic alkalosis.Under normochloremic conditions, pendrin—an apical Cl⁻/HCO₃⁻ exchanger expressed in β-intercalated cells of the cortical collecting duct—facilitates bicarbonate secretion in exchange for luminal chloride, contributing to acid–base homeostasis. During sustained chloride depletion, as observed with chronic loop or thiazide diuretic therapy, reduced luminal Cl⁻ availability limits pendrin activity, impairing bicarbonate secretion and promoting systemic metabolic alkalosis. This chloride-depletion alkalosis is mechanistically distinct from classical contraction alkalosis and may persist despite restoration of intravascular volume, underscoring chloride deficiency as the primary driver. Restoration of chloride availability (e.g. NaCl or KCl) rapidly normalises bicarbonate handling and systemic pH, even during ongoing diuresis.

Persistent chloride loss during loop or thiazide therapy promotes metabolic alkalosis and reinforces diuretic resistance by favouring bicarbonate retention and compensatory distal NaCl reabsorption [2]. This phenotype—chloride-depletion alkalosis—differs from simple volume contraction, as hypochloraemia rather than hypovolaemia is the dominant driver of alkalosis and secondary neurohormonal activation [23, 24]. Cl⁻ depletion also impairs distal acid–base handling by limiting pendrin-mediated bicarbonate secretion in the cortical collecting duct (Fig. 3), thereby sustaining alkalosis and reducing natriuretic efficiency [22–24].

**Figure 4:**
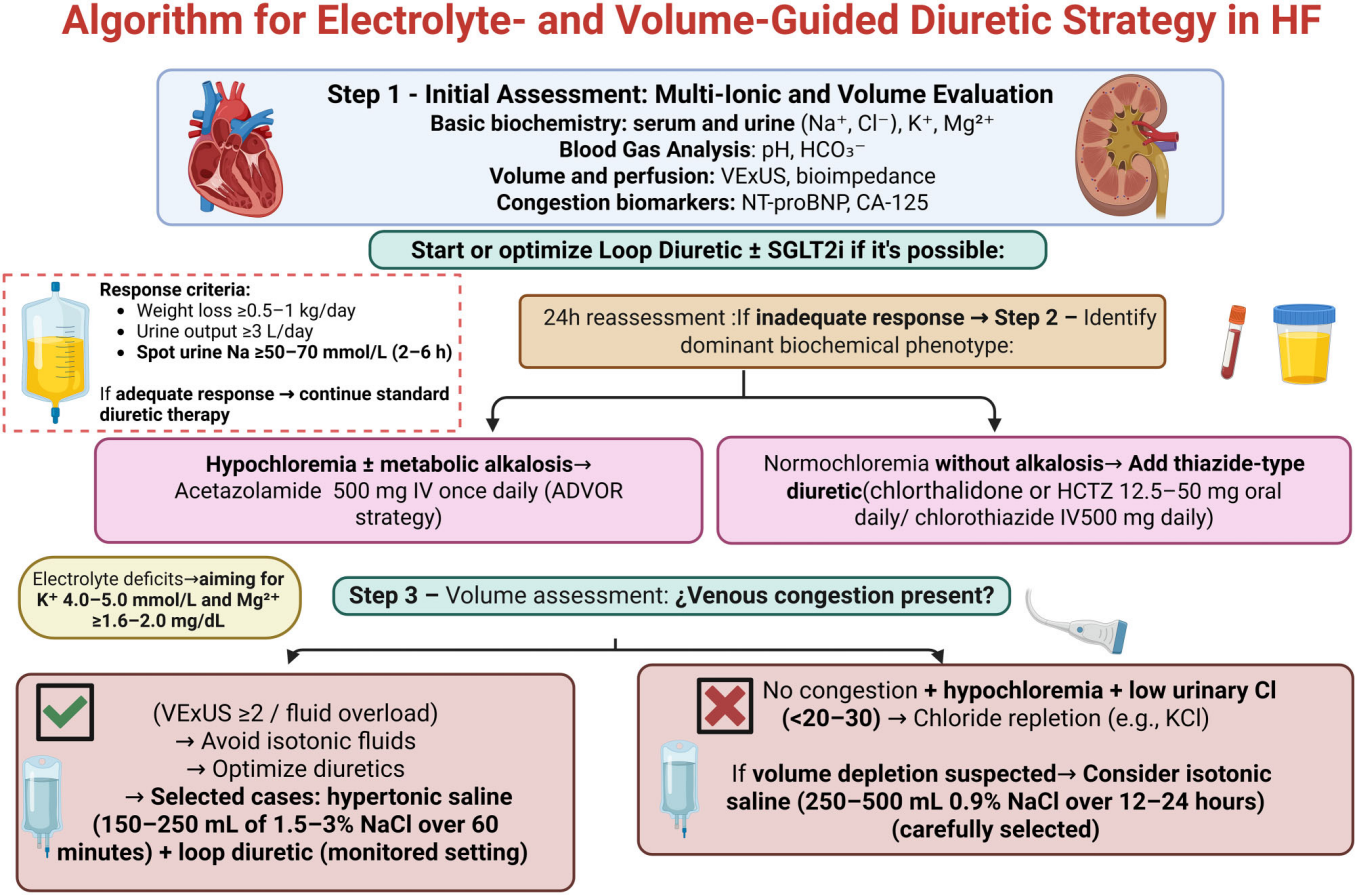
Algorithm for multi-ionic and volume-guided diuretic strategy in HF

Correction of chloride deficiency restores macula densa signalling, suppresses maladaptive RAAS activation, and improves diuretic responsiveness, in parallel with downregulation of WNK–SPAK–NCC/NKCC pathways [7, 10, 32]. Because metabolic alkalosis and hypokalaemia synergistically reduce distal sodium excretion, simultaneous correction is generally required to reverse diuretic resistance. Recent controlled data from a randomized crossover study showed that acute potassium loading produced similar intracellular uptake across interventions, but kaliuresis and distal K⁺ secretion were significantly greater with potassium citrate than with potassium chloride, differences attributable to acid–base effects rather than potassium itself; these findings reinforce that Cl⁻, bicarbonate, and potassium operate as a tightly coupled regulatory network rather than independent determinants [13].

## CENTRAL CONCEPT

Diuretic resistance emerges from coordinated disruption of chloride, potassium, and acid–base balance, and effective reversal therefore depends on simultaneous multi-ionic correction rather than focusing on sodium or chloride in isolation.

## CLINICAL EVIDENCE: ELECTROLYTE DISORDERS, DIURETIC RESISTANCE, AND PROGNOSIS IN HF

### Chloride depletion, diuretic resistance, and adverse events​

Hypochloraemia is one of the most consistent biochemical determinants of impaired diuretic response and adverse prognosis in HF [33]. Across multiple multicentre cohorts, low serum chloride identifies patients with markedly reduced natriuretic efficiency and attenuated responsiveness to loop diuretics [34–36]. In Renal Optimization Strategies Evaluation in Acute Heart Failure (ROSE-AHF), lower baseline chloride independently predicted reduced diuretic efficiency and blunted natriuresis during acute decompensation [37], and in the ALCALOTIC cohort hypochloraemia (chloride <96 mmol/l) was associated with significantly less in-hospital weight loss despite optimized loop diuretic therapy [38].

Observational analyses suggest that chloride depletion modifies the renal response to intensified diuretic strategies: in ALCALOTIC, the natriuretic benefit of sequential nephron blockade was confined to patients with normal or elevated chloride, whereas no improvement was seen in hypochloraemic individuals [38]. Dynamic studies showed that early increases in serum chloride in Saline in Acute decompensated heaRT failure (SALT-HF) were associated with greater urine output, enhanced net fluid loss, shorter time to decongestion, and lower risk of in-hospital worsening HF, and a chloride rise ≥4 mmol/l predicted more favourable short-term outcomes, linking chloride availability to renal NaCl handling and effective decongestion [39].

Serum chloride also has independent prognostic value. *Post hoc* analyses from PROTECT (hospitalized HFrEF) and TOPCAT (heart failure with preserved ejection fraction (HFpEF) outpatients) showed that hypochloraemia independently predicted higher all-cause mortality at 6 months and 5 years, respectively, after adjustment for sodium, renal function, and established risk markers [33, 40]. In ROSE-AHF, each 1 mmol/l increase in chloride was associated with a 14% reduction in 60-day mortality and a 9% reduction at 180 days [37], and a systematic review (>25 000 patients, 16 studies) confirmed consistent associations between low chloride and mortality, HF rehospitalization, impaired diuretic response, and need for advanced therapies, whereas data on hyperchloraemia were less consistent [34]. In CKD cohorts, low chloride has also been linked to atrial fibrillation, stroke, incident HF, and all-cause mortality, positioning chloride as both a pathophysiological mediator and a prognostic biomarker [41].

### Potassium and magnesium depletion, diuretic resistance, and outcomes

Potassium depletion has important prognostic and therapeutic implications. Across large registries and trials, serum potassium <4.0 mmol/l—and particularly <3.5 mmol/l—is consistently associated with higher all-cause and cardiovascular mortality and increased risk of ventricular arrhythmias and sudden cardiac death in both HFrEF and HFpEF, in acute and chronic settings [42–44]. Clinically, hypokalaemia often coexists with hypochloraemia and metabolic alkalosis, delineating a multi-ionic phenotype associated with impaired diuretic responsiveness and heightened neurohormonal activation [5, 45].

The POTCAST trial evaluated whether proactively raising plasma potassium into the high-normal range (approximately 4.5–5.0 mmol/l) using diet, supplements, and mineralocorticoid receptor antagonists could modify arrhythmic risk in patients with implantable cardioverter defibrillators and showed that targeting high-normal potassium reduced ventricular arrhythmias, appropriate implantable cardioverter-defibrillator therapies, HF hospitalizations, and all-cause mortality without a significant excess of clinically relevant dyskalaemia [46]. These findings support potassium as a modifiable determinant of arrhythmic and HF outcomes, although extrapolation to unselected HF populations should remain cautious [47].

Hypomagnesaemia is frequent in HF, especially among patients receiving loop or sequential diuretic therapy, with reported prevalence up to 17% in contemporary cohorts and higher rates in earlier HFrEF series [16, 48, 49]. Magnesium imbalance (both hypo- and hypermagnesaemia, within conventional reference ranges) reflects the combined effects of malnutrition, intestinal congestion, neurohormonal activation, diabetes, and intensive diuretic use [49], and low serum magnesium has been associated with increased HF hospitalization, arrhythmias, and mortality, particularly when coexisting with hypokalaemia or metabolic alkalosis; hypermagnesaemia in advanced renal dysfunction has also been associated with increased risk [16, 17, 50–53].

Diuretic therapy is a major contributor to magnesium depletion, as both loop and thiazide agents promote renal magnesium wasting, and chronic hypomagnesaemia often accompanies refractory hypokalaemia, complicating electrolyte correction. Hypokalaemia (<3.5 mg/dl)) and hypomagnesaemia (<1.6 mg/dl) impairs K⁺ stabilization via ROMK disinhibition and increases QT interval prolongation risk, indirectly limiting safe mineralocorticoid receptor antagonist (MRA) up-titration until corrected [16, 17]. Observational and interventional data indicate that the prognostic impact of magnesium is context-dependent: in GALACTIC-HF, hypermagnesaemia was associated with worse outcomes, probably reflecting more severe renal dysfunction [49], and PROMISE and EVEREST similarly linked higher magnesium levels to more frequent HF events and death, without a consistent independent signal for low magnesium [52, 53]. Conversely, a large Veterans Affairs cohort showed that oral magnesium supplementation in HF patients with documented hypomagnesaemia (<1.7 mg/dl) was associated with lower risk of 1-year HF hospitalization or death, particularly at levels <1.6 mg/dl, whereas supplementation in normomagnesaemic patients was neutral or unfavourable [54]. Current data support routine magnesium monitoring, targeted correction of low-range values—especially in symptomatic or arrhythmia-prone patients—and avoidance of indiscriminate supplementation at mid- or high-range levels, taking baseline magnesium and renal function into account [49].

### Metabolic alkalosis, diuretic resistance, and clinical outcomes

From an evolutionary perspective, the kidney is primarily adapted to defend against metabolic acidosis rather than alkalosis, reflecting the continuous endogenous acid load imposed by diet and metabolism, and the relative rarity of chronic alkali excess in ancestral environments. As a consequence, once metabolic alkalosis develops, renal mechanisms for bicarbonate elimination are comparatively limited, and maintenance processes (chloride and potassium depletion, mineralocorticoid excess, and reduced effective arterial blood volume) often dominate, particularly in patients receiving chronic loop or thiazide therapy [24]. In contemporary practice, metabolic alkalosis is among the most common acid–base disorders in hospitalized patients, especially in those with HF, renal insufficiency, and intensive diuretic use, and observational studies in critical care populations have shown that severe alkalosis (arterial pH above 7.55–7.60) is associated with substantially increased mortality [24]. In acute HF cohorts, metabolic alkalosis and elevated serum bicarbonate are consistently linked to impaired diuretic response, longer length of stay, and higher rates of HF readmission, although recent large registries have not demonstrated a robust association with short-term all-cause mortality [55]. These data support viewing metabolic alkalosis not merely as an epiphenomenon of diuretic therapy, but as a clinically relevant marker and potential mediator of adverse outcomes that warrants proactive identification and correction within a multi-ionic framework [38].

Metabolic alkalosis is common in both acute and chronic HF, with prevalence around 9%–12% among patients hospitalized with acute decompensated HF [45, 55]. It is most often associated with chronic or high-dose loop and thiazide therapy, which induces Cl⁻ and K⁺ depletion and activates neurohormonal pathways that favour bicarbonate retention [45, 55, 56]. Diuretic-induced hypochloraemia and hypokalaemia impair renal bicarbonate excretion and sustain maladaptive distal sodium reabsorption, thereby promoting diuretic resistance and persistent congestion [23, 34, 56].

Renal venous congestion exacerbates diuretic-induced metabolic alkalosis through three mechanisms: (i) elevated intratubular pressure impairs distal HCO₃⁻ excretion; (ii) reduced distal Cl⁻ delivery limits pendrin-mediated bicarbonate secretion; and (iii) enhanced ammoniagenesis from proximal tubule ischaemia promotes HCO₃⁻ generation, sustaining alkalosis maintenance [24, 25].

Observational studies consistently show that elevated serum bicarbonate (typically ≥28 mmol/l) is associated with impaired natriuretic response, longer hospital stay, and increased HF readmission, independent of chloride and potassium [55, 57, 58]. Persistent metabolic alkalosis has also been linked to higher HF event rates and mortality in CKD, suggesting that its adverse implications extend beyond HF-specific populations [58, 59]. In advanced CKD, metabolic alkalosis correction is challenging as CKD sustains primary alkalotic processes (diuretic- or chloride-depletion-mediated) through impaired HCO₃⁻ excretion and pendrin dysfunction, rather than causing dilutional acidosis [23]. Hypochloraemia and loop diuretic use remain dominant generation mechanisms even with estimated glomerular filtration rate (eGFR) <30 ml/min/1.73 m^2^ [23]. Interpretation of bicarbonate requires careful context, as elevated levels may reflect renal compensation for chronic hypercapnia or accumulation due to impaired renal function rather than primary metabolic alkalosis; accordingly, bicarbonate should be evaluated together with arterial blood gases, renal and respiratory function, and overall clinical status [23, 24].

Taken together, clinical evidence supports a unifying model in which electrolyte disturbances—especially hypochloraemia, hypokalaemia, magnesium abnormalities, and metabolic alkalosis—act as interconnected markers and mediators of diuretic resistance, neurohormonal activation, and adverse outcomes in HF. A multi-ionic perspective that systematically evaluates chloride, potassium, magnesium, and acid–base balance offers a more coherent framework for risk stratification and helps explain why sodium-centred strategies alone capture only a fraction of the cardiorenal burden [33–35, 41].

## IS THERE A ROLE FOR ELECTROLYTE-GUIDED DIURETIC THERAPY?

### Natriuresis-guided strategies: effective early markers, but limited alone

Urinary sodium is the best-validated biochemical marker of short-term diuretic responsiveness in acute HF. A spot urine sodium measured within 2 h of loop diuretic administration can identify inadequate natriuresis and support early therapeutic intensification [60–62]. Recent expert consensus recommends spot urinary Na⁺ targets of >50–70 mmol/l at 2 h postloop diuretic dose, with ≥70 mmol/l considered an adequate early natriuretic response to guide therapy intensification; simultaneous monitoring of urinary chloride is advised to avoid missing contraction alkalosis [63]. Randomized data from Pragmatic Urinary Sodium-based diuretic tHerapy in Acute Heart Failure show that natriuresis-guided protocols increase early natriuresis and diuresis and modestly shorten hospital stay without excess acute kidney injury or major safety concerns, although these benefits have not translated into improved longer-term outcomes such as mortality or HF rehospitalization [62, 64].

Thus, urinary sodium is a powerful operational tool for early response assessment, but its value as a sole therapeutic target appears limited. Using urinary sodium as the only decision driver may encourage aggressive up-titration of loop and loop–thiazide combinations, with attendant risks of hypokalaemia, hypochloraemia, metabolic alkalosis, and worsening kidney function, and therefore natriuretic targets should be integrated with bedside assessment and broader biochemical monitoring [5, 56, 60, 63].

### Chloride-guided therapy: essential physiology, but reductionist if isolated

Chloride has re-emerged as a key determinant of diuretic efficacy, neurohormonal activation, and cardiorenal interactions [5, 25, 65, 66]. Across multiple cohorts, low serum chloride consistently identifies patients with reduced natriuretic efficiency and attenuated response to loop diuretics, while dynamic increases in chloride parallel improved decongestion, as detailed above [5, 25, 37–39].

In SALT-HF and its chloride-focused substudy, co-administration of hypertonic saline with loop diuretics increased serum chloride within 72 h and was associated with greater cumulative urine output, enhanced net fluid loss, and shorter time to decongestion, with early rises in chloride (≥4 mmol/l) linked to lower rates of in-hospital worsening HF and favourable 90-day trends [39]. These findings are compatible with the concept that restoring chloride availability, central to distal NaCl handling, may facilitate decongestion, but remain hypothesis-generating and do not establish chloride-targeted therapy as standard of care. From a physiological standpoint, correcting hypochloraemia without careful attention to sodium load, renal reserve, or potassium status risks hyperchloraemic acidosis, volume overload, or impaired kidney perfusion [67, 68]. Given the frequent coexistence of chloride, sodium, potassium, and acid–base abnormalities, purely chloride-centric algorithms are prone to oversimplification and should instead be embedded in a broader multi-ionic framework [33, 34].

### Potassium and acid–base parameters: powerful modifiers, but weak stand-alone guides

Potassium functions primarily as a safety and prognostic biomarker, closely linked to arrhythmic risk, neurohormonal activation, and tolerance of diuretic and neurohormonal therapies [44, 69]. Hypokalaemia often accompanies heightened RAAS activity, increased tubular sodium avidity, and relative diuretic resistance, but these relationships are largely supported by mechanistic and observational data rather than prospective potassium-guided titration trials [44, 70]. Urinary potassium/creatinine ratios have not shown sufficient discriminatory capacity to guide diuretic selection [71], suggesting that potassium is best used to inform risk mitigation (electrolyte supplementation, optimization of RAAS blockade) rather than to dictate specific diuretic regimens.

Metabolic alkalosis, frequently induced by loop–thiazide combinations and exacerbated by renal venous congestion, modifies diuretic responsiveness and can identify patients who benefit from short-course acetazolamide [5, 56, 72]. ADVOR showed that adding acetazolamide to standardized high-dose loop diuretics improved decongestive success and shortened hospital stay in acute HF, with a prespecified analysis suggesting greater proportional benefit in patients with higher baseline bicarbonate, particularly ≥27 mmol/l [57, 73]. These data position elevated bicarbonate as a potentially actionable modifier of diuretic strategy in ADVOR-like settings, but evidence remains confined to this trial and *post hoc* analyses, and cannot be generalized to chronic use or broader populations; acid–base parameters have not been validated as universal titration tools, and prolonged acetazolamide therapy is not recommended [57, 72, 73].

### Bridging the gap: from physiological complexity to clinical practice

Given the intricate interplay between sodium, chloride, potassium, magnesium, and acid–base balance, decongestion cannot be safely optimized through a single-analyte lens. The limitations of current natriuresis-centric or chloride-only strategies underscore the need for a dynamic, integrated approach that acknowledges multi-ionic drivers of diuretic resistance and aims to harmonize rather than single out ionic targets.

**Step 1–Baseline and early assessment**

A multi-ionic, physiology-guided strategy (Fig. 4) begins with structured early evaluation to distinguish patients responding to standard loop diuretics from those requiring timely therapeutic escalation. This approach limits empirical dose escalation and grounds initial decisions in objective physiological and biochemical data [60, 61, 74, 75].

Loop diuretics remain the cornerstone of decongestive therapy. Concurrently, guideline-directed medical therapy should be optimized from the outset, including sodium–glucose cotransporter-2 inhibitors (SGLT2i) when renal function permits. Early SGLT2i initiation improves natriuretic efficiency and is generally not associated with clinically significant electrolyte disturbances [60, 61].

To operationalize this physiological framework clinically, we propose an operational definition of diuretic resistance based on current literature [72].

### Box 1. Operational definition of diuretic resistance

Diuretic resistance is defined as failure to achieve adequate decongestion within 24–48 h despite optimized intravenous loop diuretic therapy (appropriate dose, frequency, and correction of reversible factors).

This includes failure to meet one or more of the following targets:

- Weight loss ≥0.5–1.0 kg/day
- Urine output ≥3 l/24 h
- Adequate natriuretic response (e.g. spot urinary Na⁺ ≥50–70 mmol/l 2–6 h postloop diuretic or satisfactory 24-h natriuresis per local protocols) [62–64].

Volume status assessment should integrate clinical examination with available adjunctive tools: lung ultrasound (B-lines), venous Doppler congestion scores (e.g. VExUS), and bioimpedance or similar technologies to estimate total body fluid [5, 75].

These tools refine early decision-making by differentiating intravascular from interstitial congestion and providing dynamic markers of venous decongestion improvement (e.g. portal vein pulsatility). They also prevent inappropriate fluid restriction or volume replacement, particularly in right ventricular dysfunction where chronic right atrial pressure elevation may preclude full normalization of composite scores like VExUS [5, 75].

**Step 2–Biochemical and acid–base profiling**

Mandatory reassessment at ∼24 h is central. Clinicians should re-evaluate urine output, weight change, serum electrolytes (Na⁺, Cl⁻, K⁺, and Mg²⁺), bicarbonate, and creatinine.

Inadequate early natriuresis and minimal weight loss at 24 h predict poor decongestion and worse outcomes, prompting early diuretic strategy modification. Early chloride and bicarbonate shifts may signal emerging hypochloraemic metabolic alkalosis or other resistance patterns before overt clinical deterioration. Blind loop diuretic escalation without biochemical reassessment risks hypokalaemia, metabolic alkalosis, and renal worsening [5, 25, 60].

Early biochemical phenotyping guides phenotype-specific interventions by identifying reproducible ionic patterns reflecting predominant response-limiting mechanisms. Table 3 stratifies common profiles by serum chloride, bicarbonate/pH, potassium, and magnesium, linking each to pathophysiological interpretation and targeted therapies [5, 25, 60, 61, 75].

**Table 3:** Identify the biochemical and acid–base profile.

Congestion phenotype (haemodynamic status)	Biochemical profile	Pathophysiological interpretation	Targeted therapeutic intervention
**Predominant intravascular congestion (VExUS ≥2; dilated IVC; elevated venous pressures)**	**Hypochloraemia + metabolic alkalosis (Cl⁻ <98 mmol/l; HCO₃⁻ >28 mmol/l)**	Chloride-depletion, alkalosis-driven loop resistance; impaired distal Cl⁻ sensing; pendrin activation.	• IV Acetazolamide (500 mg) to correct alkalosis and restore Cl⁻ sensitivity. • Hypertonic saline (HSS) + loop diuretics in diuretic-resistant states.
	**Hypochloraemia without alkalosis (Cl⁻ <98 mmol/l; normal HCO₃⁻)**	Dilutional or depletional hypochloraemia in the context of congestion; not equivalent to hypovolaemia.	• Avoid isotonic volume expansion. • Optimize loop dosing and frequency. • Consider HSS if resistance persists.
**Predominant tissue congestion (VExUS 0–1; residual B-lines/edema)**	**Normochloraemia or hyperchloraemia (Cl⁻ ≥98 mmol/l)**	Refill-limited intravascular space with distal tubular adaptation (NCC upregulation).	• Sequential nephron blockade (thiazide-type diuretics). • Close BP monitoring.
**Euvolemic chloride-depleted phenotype (VExUS 0; no pulmonary congestion)**	**Hypochloraemia + preserved Na⁺ + low urinary Cl⁻ (<20–30 mmol/l)**	Sodium-avid, chloride-depleted state without overt congestion.	• Sodium-free chloride (e.g. KCl) preferred. • Isotonic saline only if strict criteria for true hypovolemia are met.
**Any congestion phenotype**	**Hypokalaemia (K⁺ <3.5 mmol/l)**	WNK–SPAK activation; arrhythmic risk; reduced diuretic efficiency.	• Up-titrate MRA (spironolactone/eplerenone). • K⁺ repletion (target 4.0–5.0 mmol/l).
	Hypomagnesaemia (Mg²⁺ <1.6 mg/dl)	ROMK disinhibition; persistent K⁺ wasting; tubular instability.	• Magnesium repletion (IV in acute setting; oral for maintenance).

HSS: hypertonic saline solution; BP: blood pressure.

The proposed sequence is:

- Identify the dominant biochemical profile, prioritizing hypochloraemia with or without metabolic alkalosis.
- Implement the corresponding targeted intervention.
- Reassess the ionic profile at 24–48 h.

Representative regimens include acetazolamide 500 mg intravenous (IV) once daily (ADVOR protocol) for hypochloraemic metabolic alkalosis or persistent alkalosis despite normochloraemia [5, 23, 57]. Thiazide-like agents may be added for inadequate natriuresis without marked chloride depletion or alkalosis: hydrochlorothiazide 12.5–25 mg orally or chlorothiazide 250–500 mg IV, daily, if gastrointestinal absorption is uncertain [25, 60].

In advanced CKD, conventional thiazides lose efficacy at low glomerular filtration rates; thiazide-like agents such as chlorthalidone or xipamide retain natriuretic activity and are preferable for sequential nephron blockade. In unstable settings, serum creatinine or eGFR may be unreliable due to non-steady-state conditions; short-term measured creatinine clearance (12–24 h) provides a more accurate dosing guide in selected patients.

Physiologically, chloride repletion enhances macula densa signalling and bicarbonate handling, while potassium/magnesium correction attenuates tubular sodium reabsorption. Combined with segment-specific diuretics, these measures overcome residual tubular resistance by aligning therapy with tubular physiology [5, 25, 27, 56].

**Step 3–Volume and dietary considerations**

Fluid and dietary strategies should be tailored to the patient’s ionic profile, hemodynamic status, and congestion phenotype rather than uniform restrictions [61, 76, 77].

Volume assessment must integrate congestion phenotypes with biochemical profiles, as venous congestion and effective intravascular depletion demand distinct approaches [78].

In persistent venous congestion (e.g. VExUS ≥2 or clear fluid overload), further isotonic volume expansion should generally be avoided. In selected diuretic-resistant cases with severe hypochloraemia, hypertonic saline co-administered with IV loop diuretics has been linked to improved diuretic efficiency and decongestion in observational cohorts and small series, including the SALT-HF experience [39].

Here, hypertonic saline (150–250 ml of 1.5%–3% NaCl over 30–60 min with furosemide) may be considered in carefully selected, monitored patients. Proposed mechanisms include osmotic extravascular fluid mobilization, enhanced renal perfusion, and reduced maladaptive sodium retention [79].

This approach is not standard care and should be confined to highly selected patients in monitored settings (e.g. step-down or intensive care unit), with close hemodynamic and biochemical surveillance. Avoid in hypervolemic or anuric patients on unmonitored wards due to risks of worsening congestion, electrolyte imbalance, or instability [79].

Conversely, in patients lacking objective venous congestion (VExUS 0, no pulmonary congestion) with hypochloraemia and low urinary chloride (<20–30 mmol/l), sodium-free chloride supplementation (e.g. potassium chloride) is preferred [5].

Isotonic saline (250–500 ml 0.9% NaCl over 12–24 h) may be considered for hypochloraemia without metabolic alkalosis, accompanied by effective volume depletion signs or diuretic intolerance, provided serum Na⁺ ≤145 mmol/l [60, 80, 81].

Although recent experimental data suggest controlled sodium chloride during protocolized diuresis modulates sodium avidity, isotonic saline lacks sufficient support for routine use beyond selected phenotypes [82].

In hypervolemia or dilutional hyponatremia, moderate sodium restriction (∼2.5–3 g NaCl/day) is reasonable; strict fluid restriction should be reserved for marked hyponatremia [61, 76, 77].

Dietary potassium and magnesium balance warrants attention, as deficits raise arrhythmic risk and impair decongestion. Maintain K⁺ at 4.0–5.0 mmol/l—especially during sequential nephron blockade—via diet and oral/IV supplementation as needed [60, 61, 83].

Typical strategies: oral potassium chloride (40–100 milliequivalents (mEq)/day) or IV (20–40 mEq over 1–2 h monitored). Correct magnesium deficits with oral supplexmentation (400–800 mg/day) for mild cases or IV magnesium sulphate (1–2 g over 1–2 h) for severe, dose-adjusted to renal function [84].

## CONCLUSIONS

Chloride has emerged as a pivotal regulator of diuretic responsiveness, acid–base balance, and neurohormonal activation, but the growing body of evidence indicates that a chloride-only perspective risks oversimplifying a complex ionic network. HF decongestion should instead be guided by an integrated, multi-ionic perspective that recognizes the interdependence of sodium, chloride, potassium, magnesium, and bicarbonate in determining natriuretic efficiency, safety, and treatment tolerance [5].

Future strategies should prioritize restoring global electrolyte harmony rather than correcting isolated abnormalities. A physiology-guided, patient-specific approach—balancing sodium restriction, preserving chloride and potassium, and ensuring adequate magnesium repletion—may better sustain diuretic efficacy, minimize neurohormonal activation, and prevent metabolic and electrical instability [5, 25].

Thus, although chloride represents an important ‘key maker’ in overcoming diuretic resistance, it should be understood as one element within a broader framework of electrolyte and volume homeostasis. Integrative corrective electrolyte strategies that simultaneously address multiple ionic pathways are likely to provide more durable and physiologically coherent decongestion in HF and align with the emerging paradigm of multi-ionic, personalized care [5, 25].

## Data Availability

No new datasets were generated or analysed in this study. This article is a narrative review based on previously published literature. Any clinical information included is derived from a single illustrative case and has been fully anonymized in accordance with applicable ethical standards. Further details are not available to protect patient confidentiality.
